# The GIST of it all: management of gastrointestinal stromal tumors (GIST) from the first steps to tailored therapy. A bibliometric analysis

**DOI:** 10.1007/s00423-024-03271-6

**Published:** 2024-03-14

**Authors:** Julian Musa, Sarah M. Kochendoerfer, Franziska Willis, Christine Sauerteig, Jonathan M. Harnoss, Ingmar F. Rompen, Thomas G. P. Grünewald, Mohammed Al-Saeedi, Martin Schneider, Julian-C. Harnoss

**Affiliations:** 1 Department of General, Visceral, and Transplantation Surgery, University Hospital Heidelberg, Im Neuenheimer Feld 420, 69120 Heidelberg, Germany; 2 Department of General, Visceral, Thoracic, and Transplantation Surgery, University Hospital Giessen and Marburg, Giessen, Germany; 3 Division of Translational Pediatric Sarcoma Research (B410), German Cancer Research Center (DKFZ), Heidelberg, Germany; 4 Hopp-Children’s Cancer Center (KiTZ), Heidelberg, Germany; 5 Institute of Pathology, University Hospital Heidelberg, Heidelberg, Germany

**Keywords:** Gastrointestinal stromal tumor, GIST, Bibliometry, Imatinib, Tyrosine kinase inhibitor, TKI

## Abstract

**Purpose:**

Improvement of patient care is associated with increasing publication numbers in biomedical research. However, such increasing numbers of publications make it challenging for physicians and scientists to screen and process the literature of their respective fields. In this study, we present a comprehensive bibliometric analysis of the evolution of gastrointestinal stromal tumor (GIST) research, analyzing the current state of the field and identifying key open questions going beyond the recent advantages for future studies to assess.

**Methods:**

Using the Web of Science Core Collection, 5040 GIST-associated publications in the years 1984–2022 were identified and analyzed regarding key bibliometric variables using the Bibliometrix R package and VOSviewer software.

**Results:**

GIST-associated publication numbers substantially increased over time, accentuated from year 2000 onwards, and being characterized by multinational collaborations. The main topic clusters comprise surgical management, tyrosine kinase inhibitor (TKI) development/treatment, diagnostic workup, and molecular pathophysiology. Within all main topic clusters, a significant progress is reflected by the literature over the years. This progress ranges from conventional open surgical techniques over minimally invasive, including robotic and endoscopic, resection techniques to increasing identification of specific functional genetic aberrations sensitizing for newly developed TKIs being extensively investigated in clinical studies and implemented in GIST treatment guidelines. However, especially in locally advanced, recurrent, and metastatic disease stages, surgery-related questions and certain specific questions concerning (further-line) TKI treatment resistance were infrequently addressed.

**Conclusion:**

Increasing GIST-related publication numbers reflect a continuous progress in the major topic clusters of the GIST research field. Especially in advanced disease stages, questions related to the interplay between surgical approaches and TKI treatment sensitivity should be addressed in future studies.

**Supplementary Information:**

The online version contains supplementary material available at 10.1007/s00423-024-03271-6.

## Introduction

Gastrointestinal stromal tumors (GIST) are malignant mesenchymal tumors deriving from lineage cells of interstitial cells of Cajal (ICC) with an annual incidence of approximately 1.2 per 10^5^ individuals [1]. Most frequently observed locations are stomach (60–65%), small intestine (20–35%), and rectum (3–5%) [1, 2]. The mainstay of GIST therapy in localized setting is surgery [1, 2], whereby main risk factors for relapse are tumor size, mitotic index, non-gastric site, and tumor rupture [1, 3]. GIST typically are resistant to conventional chemotherapy. Around 80% of GIST show varying *KIT* or *PDGFRA* mutations sensitizing for treatment with the tyrosine kinase inhibitor (TKI) imatinib [1, 4–8]. Imatinib-resistance mediating additional mutations in *KIT* or *PDGFRA* or mutations in other genes which might not sensitize for imatinib therapy are observed in a lower frequency; however, in such cases, therapy using other TKIs (such as sunitinib, regorafenib, ripretinib, avapritinib, larotrectinib, or entrectinib) might still be applicable depending on the mutational spectrum of the respective individual tumor [1, 9–13]. Patients harboring TKI-sensitizing mutations with high risk for relapse or patients in a primary metastatic setting receive (adjuvant) TKI treatment [1, 2, 14]. Neo-adjuvant TKI treatment might be considered in case of locally advanced disease to reduce tumor size and to remove the tumor with less extensive surgery [9].

While localized low-risk GIST are often curable with complete tumor resection, localized operable high-risk GIST with 3 years of adjuvant imatinib treatment after surgery show 5- and 10-year recurrence-free survival rates of 71.4% and 52.5% as well as 5- and 10-year overall survival rates of 92.0% and 79.0%, respectively [14]. Primary metastatic GIST under imatinib treatment show estimated 10-year progression-free survival rates of 10% and 10-year overall survival rates of 20% [15].

Tremendously increasing research activity as represented by increasing publication numbers in biomedical research led to substantial improvements in patient care and outcome over the recent years, but also confronts scientists with the problem of integratively processing a vast number of relevant published studies in their research fields. Bibliometric analyses can help scientists to more precisely define the present research state in a certain field and, even more important, to identify open questions and important topics that need to be addressed in future studies in order to continue to make significant progress in the field.

However, although many achievements significantly improving patient outcome have been made over the recent years, many aspects of GIST treatment, especially in the advanced/metastatic or recurrent disease setting, remain unclear. In this study, we present a comprehensive bibliometric analysis illustrating the developments and achievements in GIST research over the recent years, but also identifying unanswered questions that need to be addressed in future studies to further improve GIST patient outcome. This bibliometric analysis aims to provide a thorough and efficient overview of the GIST field for the reader, especially those that are new to the field, based on quantitative comprehensive publication-related data.

## Methods

Scientific publications focusing on GIST were extracted from the Clarivate Web of Science (WoS) Core Collection database using the title search term “Gastrointestinal Stromal Tumor* OR GIST (Title)” on the 16th of March 2023. The search was limited to publications until the 31st of December 2022. Only articles and reviews in English were considered. Since the search term “GIST” reveals several non-specific search results especially in the fields of neurology and psychiatry/psychology as well as informatic data processing (because of its meaning “main message, quintessence, summary” in English language), WoS Citations Topics Mesos regarding non-medical fields as well as neurology- and psychiatry/psychology-associated fields were excluded to increase specificity of the search results. The specified search revealed 5040 publications. Full associated bibliometric data was downloaded from the WoS database and analyzed using the R package Bibliometrix [16] (v4.0.1, open-source software, Massimo Aria and Corrado Cuccurullo, Università degli Studi di Napoli Federico II and Università degli studi della Campania Luigi Vanvitelli, Italy) including the BiblioShiny [16] interface (open-source software, Massimo Aria and Corrado Cuccurullo, Università degli Studi di Napoli Federico II and Università degli studi della Campania Luigi Vanvitelli, Italy) in RStudio (v2022.07.1, build 554, Posit PBC, Boston, USA).

For co-occurrence network analysis, VOSviewer (v1.6.18, open-source software, Nees Jan van Eck and Ludo Waltman, Leiden University, The Netherlands) software [17–19] was used. For keyword analysis using the VOSviewer software, author keywords and keywords-plus (the latter as defined by the Web of Science database: words or phrases that frequently appear in the titles of an article’s references, but do not appear in the title of the article) were considered with a minimum number of 30 occurrences. For analysis of co-occurring terms in titles and abstracts using the VOSviewer software, the binary counting method was used considering items with a minimum of 20 item occurrences and, among those, items included in the top 60% according to the calculated relevance score.

For data visualization, the Bibliometrix/BiblioShiny package [16] (v4.0.1, open-source software, Massimo Aria and Corrado Cuccurullo, Università degli Studi di Napoli Federico II and Università degli studi della Campania Luigi Vanvitelli, Italy) in RStudio (v2022.07.1, build 554, Posit PBC, Boston, USA), GraphPad Prism (v9, Dotmatics, Boston, USA), VOSviewer [17–19] (v1.6.18, open-source software, Nees Jan van Eck and Ludo Waltman, Leiden University, The Netherlands), and the web-interface Multiple List Comparator from Molbiotools (open-source online-interface software, https://molbiotools.com, as accessed on the 16th of March 2023) were used.

## Results

### General scientific activity

**Fig. 1:**
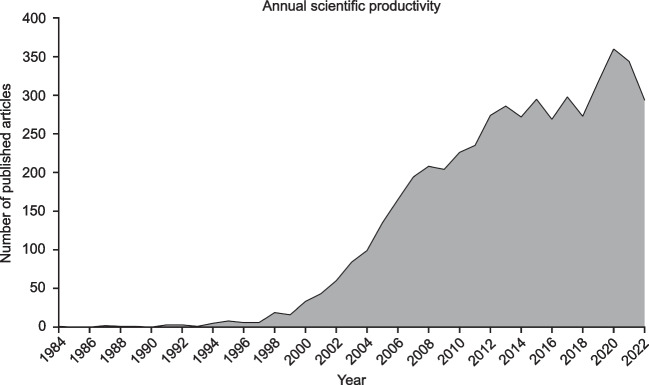
Annual scientific productivity regarding GIST-related studies as indicated by number of published articles (years 1984–2022)

In total, 5040 publications (only considering articles and reviews) in English related to GIST were identified in the time period 1984–2022. In 1984, the first GIST-specific study was published. General information about included studies are given in Supplementary Table 1. The number of GIST-related publications per year constantly increased over time with a prominent increase from year 2000 on (Fig. 1, Supplementary Table 1). The annual growth rate is 6.61% with in average 29.62 citations per publication.

### Most productive countries and collaborations

**Fig. 2:**
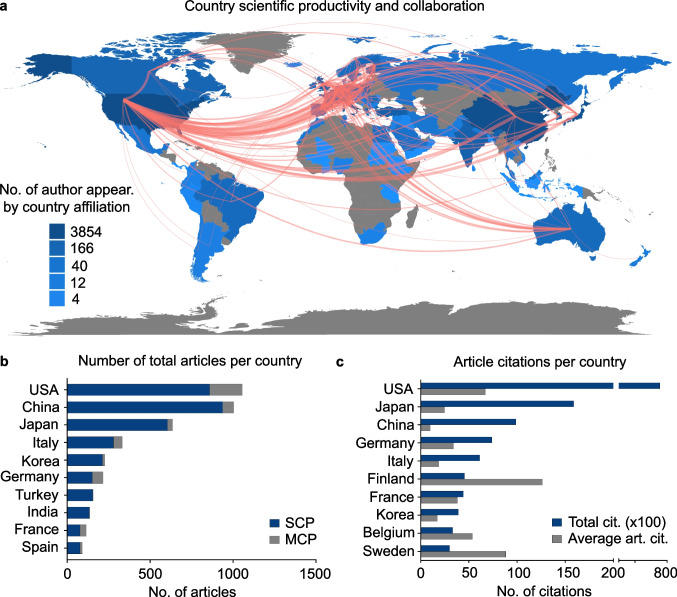
Scientific productivity regarding studies related to GIST per country. **a** Country scientific production as determined by number of author appearances by country affiliation. Red lines indicate collaboration frequencies between countries. **b** Total number of articles published per country. Country assignment was performed according to the affiliation of the corresponding author. SCP, single country publications; MCP, multiple country publications. **c** Total and average citation rates of articles per country. Country assignment was performed according to the affiliation of the corresponding author

In terms of scientific productivity per country regarding GIST research, as measured by number of author appearances per country affiliation, USA, China, Japan, Italy, and Germany show the highest scientific productivity (Fig. 2a, Supplementary Table 2). Most frequent collaborations were formed between USA-Germany, USA-China, USA-Italy, USA-Belgium, and USA-Finland (Fig. 2a, Supplementary Table 2). Regarding the number of total publications in the GIST research field, highest total article numbers show USA, China, Japan, Italy, and Korea, whereby country assignment was determined according to the affiliation of the corresponding author (Fig. 2b, Supplementary Table 2). Concerning citation numbers of published articles, USA, Japan, China, Germany, and Italy are the top countries in terms of total citations, whereby Finland, Iceland, Sweden, USA, and Belgium are the top countries in respect of average article citations (Fig. 2c, Supplementary Table 2).

### Most relevant authors/journals and their research focus

Most productive authors in respect of total number of published articles (without considering author position) in the GIST field are Heinrich M. C., Blay J. Y., Hirota S., Fletcher J. A., and Nishida T. (Table 1, Supplementary Table 3). Most cited publications (total citations and average citations per year) are given in Table 2 and Supplementary Table 3. The journals publishing most of the studies in the GIST research field are shown in Table 3 and Supplementary Table 3.

**Table 1:** Most relevant authors as indicated by the number of article publications in the GIST field

Rank	Author	No. of articles
1	Heinrich MC	85
2	Blay JY	84
3	Hirota S	83
4	Fletcher JA	80
5	Nishida T	76
6	Demetri GD	75
7	Miettinen M	74
8	Pantaleo MA	69
9	Trent JC	66
10	von Mehren M	65

**Table 2:** Most relevant articles related to GIST by total citations and total citations per year

Rank	Total citations		Total citations per year	
1	Hirota S, 1998, SCIENCE	3264	Demetri GD, 2002, NEW ENGL J MED	146.05
2	Demetri GD, 2002, NEW ENGL J MED	3213	Hirota S, 1998, SCIENCE	125.54
3	Fletcher CDM, 2002, HUM PATHOL	2183	Fletcher CDM, 2002, HUM PATHOL	99.23
4	Dematteo RP, 2000, ANN SURG	1801	Heinrich MC, 2003, SCIENCE	84.14
5	Heinrich MC, 2003, SCIENCE	1767	Heinrich MC, 2003, J CLIN ONCOL	82.43
6	Heinrich MC, 2003, J CLIN ONCOL	1731	Dematteo RP, 2000, ANN SURG	75.04
7	Joensuu H, 2001, NEW ENGL J MED	1489	Miettinen M, 2006, SEMIN DIAGN PATHOL	70
8	Miettinen M, 2006, SEMIN DIAGN PATHOL	1260	Choi H, 2007, J CLIN ONCOL	65.59
9	Miettinen M, 2001, VIRCHOWS ARCH	1245	Joensuu H, 2001, NEW ENGL J MED	64.74
10	Kindblom LG, 1998, AM J PATHOL	1203	Miettinen M, 2006, ARCH PATHOL LAB MED	55

**Table 3:** Most relevant journals publishing GIST-related articles by total number of GIST-related publications

Rank	Journal	No. of articles
1	World Journal of Gastroenterology	121
2	Clinical Cancer Research	84
3	Annals of Surgical Oncology	78
4	International Journal of Surgery Case Reports	77
5	World Journal of Surgical Oncology	62
6	Oncology Letters	61
7	American Journal of Surgical Pathology	60
8	Medicine	56
9	Hepato-gastroenterology	55
10	Human pathology	54

### Most relevant keywords and terms

**Fig. 3:**
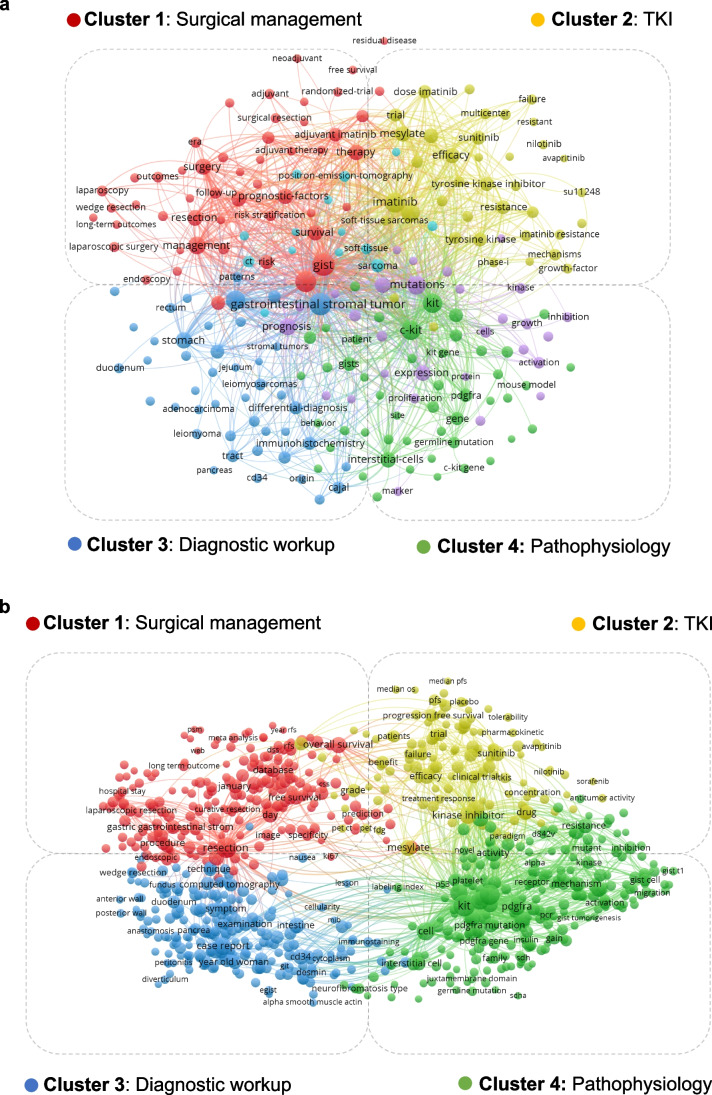
Most prominent keywords (including author keywords and keywords-plus) (**a**) and most prominent co-occurring terms in titles and abstracts (**b**) as illustrated by co-occurrence network analysis. TKI = Tyrosine kinase inhibitor

Among the most frequently occurring author keywords and keywords-plus are terms such as “Imatinib,” “(C-)KIT,” “PDGFRA,” “Sunitinib,” “Mutations,” “Diagnosis,” and “Prognosis” (Table 4, Supplementary Table 4). Most prominent keywords (including author keywords and keywords-plus) were illustrated by a co-occurrence network analysis using the VOSviewer software (Fig. 3a). Additionally, a co-occurrence network analysis was conducted using terms occurring in titles and abstracts of all included studies using the VOSviewer software (Fig. 3b). In synopsis, both co-occurrence network analyses show four major topic clusters which are associated with surgical management and TKI treatment including development/characterization of novel inhibitors, diagnostic workup, and molecular pathophysiology of GIST (Fig. 3a, b). When analyzing the most frequently occurring keywords over time separately for the time spans 1984–2000 (105 publications), 2001–2010 (1418 publications), 2011–2016 (1631 publications), and 2017–2022 (1886 publications), trends regarding all four major topic clusters as described above are evident: in the topic cluster of surgical management over time, increasingly minimally invasive approaches are represented in the literature, starting from open surgery over laparoscopic surgery to endoscopic resection techniques such as endoscopic submucosal dissection or endoscopic full-thickness resection (Fig. 3a, b, Supplementary Figure 1, Supplementary Table 4). However, although thoroughly and increasingly dealing with minimally invasive approaches, the explicit role of surgery for multiple recurrences, extended multivisceral resections in locally advanced disease, and resection of metastases, especially depending on TKI-sensitivity of the tumor, is not extensively represented in the literature (Fig. 3a, b, Supplementary Figure 1, Supplementary Table 4). In the TKI cluster, an increasing number of novel TKIs with respective clinical trials are represented in the literature (Fig. 3a, b, Supplementary Figure 1, Supplementary Table 4). In the topic clusters of molecular pathophysiology and diagnostic workup, increasing identification of specific mutations in certain genes with varying pathophysiological roles were analyzed. With that, more specific molecular diagnostic approaches to stratify patients into more specific treatment groups of TKIs were developed (Fig. 3a, b, Supplementary Figure 1, Supplementary Table 4). However, although the topic clusters 2–4 thoroughly deal with personalization of TKI therapy according to the mutational profile of the tumor especially under conditions of primary TKI therapy resistance, addressing the low percentage of remaining TKI-resistant GIST remains a main future issue to be addressed in clinical studies yet being infrequently addressed in the literature (Fig. 3a, b, Supplementary Figure 1, Supplementary Table 4). Also, potential individualization of treatment regimens of specific TKIs, as well as the potential influence of genetic germline variants on TKI resistance is not yet extensively represented in the literature and might be addressed in future studies (Fig. 3a, b, Supplementary Figure 1, Supplementary Table 4). Since relatively favorable 5- and 10-year overall survival rates are reached at least in a local disease setting [14], and since progression-free survival rates are lower than overall survival rates [14, 15] in the context of increasing minimally invasive and tailored therapy approaches, quality of life during and post GIST treatment comes into focus and is not yet majorly reflected in the literature, especially regarding surgery-related quality of life, being a further issue to be addressed in future studies (Fig. 3a, b, Supplementary Figure 1, Supplementary Table 4).

**Table 4:** Most frequently occurring author keywords and keywords-plus in GIST articles (keywords including the Web of Science search terms were excluded)

Rank	Author keywords	No. of articles	Keywords-plus	No. of articles
1	IMATINIB	594	DIAGNOSIS	838
2	KIT	336	C-KIT	797
3	PROGNOSIS	264	MUTATIONS	775
4	SURGERY	195	IMATINIB MESYLATE	652
5	IMATINIB MESYLATE	185	MANAGEMENT	630
6	IMMUNOHISTOCHEMISTRY	174	KIT	598
7	C-KIT	166	IMATINIB	545
8	SUNITINIB	159	MESYLATE	397
9	PDGFRA	158	EXPRESSION	389
10	STOMACH	135	PROGNOSTIC-FACTORS	388

## Discussion

The here-presented comprehensive bibliometric analysis depicts the landscape of GIST-associated literature throughout the past decades since GIST became an independent topic in 1984. The increasing number of publications associated with GIST from year 2000 onwards might to some extent be related to a general increase of publication numbers in the biomedical field [20, 21], but also might be majorly determined by the FDA approval of imatinib in 2001 with respective long-term follow-up studies and consecutive development and clinical testing of further TKIs [1, 2, 6–8, 10, 12, 14, 15]. Also increasing usage of minimally invasive surgical techniques, including robotic and endoscopic resection techniques, whose feasibility is also at least in part related to the efficacy of TKI treatment, might as well account for increasing publication numbers from the year 2000 onwards [22–27].

Associated with the increasing GIST-related research activity as represented by increasing number of GIST-related publications, overall survival of GIST patients significantly increased over time, especially with increasing availability of specific TKIs [28]. However, using multimodal treatment, patient outcome of localized low- and high-risk GIST is relatively favorable by now [14], whereas primary metastatic GIST still show estimated 10-year progression-free survival rates of 10% and 10-year overall survival rates of 20% under imatinib treatment [15], leaving room for improvement.

However, although significant achievements were made in many aspects of GIST management, especially in such advanced disease settings, many conditions are not yet definitive. Regarding surgical therapy, the role of metastasis or recurrence resection in TKI-sensitive GIST is not clear: only a single prospective randomized trial was conducted which was closed early due to poor accrual, suggesting a benefit from residual disease surgery (2-year progression-free survival 88.4% in the surgery plus imatinib arm vs 57.7% in the imatinib alone arm) although the results were not statistically significant [29]. Data from retrospective studies support these results reporting an oncological benefit of resection for residual metastatic/recurrent and focally progressive lesions under TKI treatment [1, 2, 30–32]. The explicit role of surgery for multiple recurrences and extended multivisceral resections in locally advanced disease has not yet been sufficiently investigated in TKI-sensitive as well as TKI-insensitive GIST [33–35]. Thus, especially in locally advanced, recurrent, and metastatic disease stages, questions related to the interplay between surgical approaches and TKI treatment sensitivity need to be addressed in future studies.

TKI resistance, no matter if due to TKI-insensitive mutational profile of the tumor (such as so-called wild-type GIST harboring no *KIT* or *PDGFRA* mutation) or acquired after/under TKI therapy, is a clinical challenge for the therapy of advanced, recurrent, and metastatic GIST [1, 2, 36, 37]. Additionally, TKI response might as well depend on genetic germline polymorphisms influencing therapy response and risk of disease progression under TKI therapy [38]. Although imatinib alternatives in GIST with a primarily imatinib-resistant molecular profile (e.g., avapritinib treatment in *PDGFRA* D842V–mutant GIST) and second, third, or fourth line TKIs in GIST with primarily imatinib-sensitive molecular profile that developed imatinib-resistance under imatinib-treatment (e.g., sunitinib, regorafenib, or ripritinib) are meanwhile available and effective depending on the specific respective mutational tumor profile [1, 2, 39, 40], novel strategies for the low percentage of residual resistant GIST need to be developed. Additionally, personalization of treatment regimens of specific TKIs appears to be important in improving individualized GIST treatment [41].

However, since relatively favorable 5- and 10-year overall survival rates are reached at least in local low- and high-risk disease settings [14] and since increasingly minimally invasive surgical therapies and tailored TKI treatments are applied [1, 2], quality of life during and post GIST treatment comes into focus. Especially, since progression-free survival rates are lower than overall survival rates [14, 15], assessment of quality of life under and after therapy appears to be even more important. Quality of life with respect to TKI treatment of GIST patients has already been assessed in several studies [42–46], whereby surgery-related quality of life, including surgery in an advanced disease setting, needs to be determined in future studies.

## Conclusion

In conclusion, by performing a comprehensive bibliometric analysis illustrating the developments and achievements in GIST research over the recent years, we show that increasing GIST-related publication numbers reflect a continuous progress in the major topic clusters of the GIST research field. However, although many achievements significantly improving patient outcome have been made over the recent years, especially in advanced, recurrent, and metastatic disease stages surgery-related questions and certain specific questions concerning (further-line) TKI treatment resistance need to be addressed in future studies. In this regard, especially the interplay between surgical approaches and TKI treatment sensitivity should be considered.

### Supplementary Information

Below is the link to the electronic supplementary material.

Supplementary file1 (PDF 7050 KB)

Supplementary file2 (XLSX 53 KB)

## Data Availability

No datasets were generated or analyzed during the current study.

## References

[1] Blay JY, Kang YK, Nishida T, von Mehren M (2021). Gastrointestinal stromal tumours. Nat Rev Dis Primers.

[2] Casali PG, Blay JY, Abecassis N (2022). Gastrointestinal stromal tumours: ESMO-EURACAN-GENTURIS Clinical Practice Guidelines for diagnosis, treatment and follow-up. Ann Oncol.

[3] Joensuu H, Vehtari A, Riihimäki J (2012). Risk of recurrence of gastrointestinal stromal tumour after surgery: an analysis of pooled population-based cohorts. Lancet Oncol.

[4] Hirota S, Isozaki K, Moriyama Y (1998). Gain-of-function mutations of c-kit in human gastrointestinal stromal tumors. Science.

[5] Wozniak A, Rutkowski P, Piskorz A (2012). Prognostic value of KIT/PDGFRA mutations in gastrointestinal stromal tumours (GIST): Polish Clinical GIST Registry experience. Ann Oncol.

[6] Heinrich MC, Owzar K, Corless CL (2008). Correlation of kinase genotype and clinical outcome in the North American intergroup phase III trial of imatinib mesylate for treatment of advanced gastrointestinal stromal tumor: CALGB 150105 study by Cancer and Leukemia Group B and Southwest Oncology Group. J Clin Oncol.

[7] Debiec-Rychter M, Sciot R, Le Cesne A (2006). KIT mutations and dose selection for imatinib in patients with advanced gastrointestinal stromal tumours. Eur J Cancer.

[8] Gastrointestinal Stromal Tumor Meta-Analysis Group (MetaGIST) (2010). Comparison of two doses of imatinib for the treatment of unresectable or metastatic gastrointestinal stromal tumors: a meta-analysis of 1,640 patients. J Clin Oncol.

[9] von Mehren M, Joensuu H (2018). Gastrointestinal stromal tumors. J Clin Oncol.

[10] Heinrich MC, Maki RG, Corless CL (2008). Primary and secondary kinase genotypes correlate with the biological and clinical activity of sunitinib in imatinib-resistant gastrointestinal stromal tumor. J Clin Oncol.

[11] Evans EK, Gardino AK, Kim JL (2017). A precision therapy against cancers driven by KIT/PDGFRA mutations. Sci Transl Med.

[12] Smith BD, Kaufman MD, Lu WP (2019). Ripretinib (DCC-2618) is a switch control kinase inhibitor of a broad spectrum of oncogenic and drug-resistant KIT and PDGFRA variants. Cancer Cell.

[13] Klug LR, Khosroyani HM, Kent JD, Heinrich MC (2022). New treatment strategies for advanced-stage gastrointestinal stromal tumours. Nat Rev Clin Oncol.

[14] Joensuu H, Eriksson M, Sundby Hall K (2020). Survival outcomes associated with 3 years vs 1 year of adjuvant imatinib for patients with high-risk gastrointestinal stromal tumors: an analysis of a randomized clinical trial after 10-year follow-up. JAMA Oncol.

[15] Casali PG, Zalcberg J, Le Cesne A (2017). Ten-year progression-free and overall survival in patients with unresectable or metastatic GI stromal tumors: long-term analysis of the European Organisation for Research and Treatment of Cancer, Italian Sarcoma Group, and Australasian Gastrointestinal Trials Group intergroup phase III randomized trial on imatinib at two dose levels. J Clin Oncol.

[16] Aria M, Cuccurullo C (2017). bibliometrix: An R-tool for comprehensive science mapping analysis. J Informet.

[17] van Eck NJ, Waltman L (2010). Software survey: VOSviewer, a computer program for bibliometric mapping. Scientometrics.

[18] Waltman L, van Eck NJ, Noyons ECM (2010). A unified approach to mapping and clustering of bibliometric networks. J Informetr.

[19] Perianes-Rodriguez A, Waltman L, van Eck NJ (2016). Constructing bibliometric networks: a comparison between full and fractional counting. J Informetr.

[20] Bornmann L, Haunschild R, Mutz R (2021). Growth rates of modern science: a latent piecewise growth curve approach to model publication numbers from established and new literature databases. Humanit Soc Sci Commun.

[21] Bornmann L, Mutz R (2015). Growth rates of modern science: a bibliometric analysis based on the number of publications and cited references. J Assoc Inf Sci Technol.

[22] Wang C, Gao Z, Shen K (2020). Safety and efficiency of endoscopic resection versus laparoscopic resection in gastric gastrointestinal stromal tumours: a systematic review and meta-analysis. Eur J Surg Oncol.

[23] De Vogelaere K, Van Loo I, Peters O, Hoorens A, Haentjens P, Delvaux G (2012). Laparoscopic resection of gastric gastrointestinal stromal tumors (GIST) is safe and effective, irrespective of tumor size. Surg Endosc.

[24] Shi F, Li Y, Pan Y (2019). Clinical feasibility and safety of third space robotic and endoscopic cooperative surgery for gastric gastrointestinal stromal tumors dissection : a new surgical technique for treating gastric GISTs. Surg Endosc.

[25] Ye X, Kang WM, Yu JC, Ma ZQ, Xue ZG (2017). Comparison of short- and long-term outcomes of laparoscopic vs open resection for gastric gastrointestinal stromal tumors. World J Gastroenterol.

[26] Lin J, Huang C, Zheng C (2014). Laparoscopic versus open gastric resection for larger than 5 cm primary gastric gastrointestinal stromal tumors (GIST): a size-matched comparison. Surg Endosc.

[27] Ceccarelli G, Costa G, De Rosa M (2021). Minimally invasive approach to gastric GISTs: analysis of a multicenter robotic and laparoscopic experience with literature review. Cancers.

[28] Call JW, Wang Y, Montoya D, Scherzer NJ, Heinrich MC (2019). Survival in advanced GIST has improved over time and correlates with increased access to post-imatinib tyrosine kinase inhibitors: results from Life Raft Group Registry. Clin Sarcoma Res.

[29] Du CY, Zhou Y, Song C (2014). Is there a role of surgery in patients with recurrent or metastatic gastrointestinal stromal tumours responding to imatinib: a prospective randomised trial in China. Eur J Cancer.

[30] Bauer S, Rutkowski P, Hohenberger P (2014). Long-term follow-up of patients with GIST undergoing metastasectomy in the era of imatinib – analysis of prognostic factors (EORTC-STBSG collaborative study). Eur J Surg Oncol.

[31] Mussi C, Ronellenfitsch U, Jakob J (2010). Post-imatinib surgery in advanced/metastatic GIST: is it worthwhile in all patients?. Ann Oncol.

[32] Raut CP, Posner M, Desai J (2006). Surgical management of advanced gastrointestinal stromal tumors after treatment with targeted systemic therapy using kinase inhibitors. J Clin Oncol.

[33] Wong JSM, Tan GHC, Quek R (2017). Is multivisceral resection in locally advanced gastrointestinal stromal tumours an acceptable strategy?. ANZ J Surg.

[34] Racz JM, Sunil S, Abramowitz D (2015). Multivisceral resections for gastrointestinal stromal tumors: are the risks justifiable?. Surg Oncol.

[35] Schurr P, Kohrs D, Reichelt U (2009). Repeated surgery improves survival in recurrent gastrointestinal stromal tumors: a retrospective analysis of 144 patients. Dig Surg.

[36] Napolitano A, Vincenzi B (2019). Secondary KIT mutations: the GIST of drug resistance and sensitivity. Br J Cancer.

[37] Kays JK, Sohn JD, Kim BJ, Goze K, Koniaris LG (2018). Approach to wild-type gastrointestinal stromal tumors. Transl Gastroenterol Hepatol.

[38] Ravegnini G, Urbini M, Simeon V (2019). An exploratory study by DMET array identifies a germline signature associated with imatinib response in gastrointestinal stromal tumor. Pharmacogenomics J.

[39] Rizzo A, Pantaleo MA, Astolfi A, Indio V, Nannini M (2021). The identity of PDGFRA D842V-mutant gastrointestinal stromal tumors (GIST). Cancers (Basel).

[40] Heinrich MC, Jones RL, von Mehren M (2020). Avapritinib in advanced PDGFRA D842V-mutant gastrointestinal stromal tumour (NAVIGATOR): a multicentre, open-label, phase 1 trial. Lancet Oncol.

[41] Nannini M, Nigro MC, Vincenzi B (2017). Personalization of regorafenib treatment in metastatic gastrointestinal stromal tumours in real-life clinical practice. Ther Adv Med Oncol.

[42] van de Wal D, Elie M, Le Cesne A (2022). Health-related quality of life and side effects in gastrointestinal stromal tumor (GIST) patients treated with tyrosine kinase inhibitors: a systematic review of the literature. Cancers (Basel).

[43] Heinrich MC, George S, Zalcberg JR (2020). Quality of life (QoL) and self-reported function with ripretinib in ≥4th-line therapy for patients with gastrointestinal stromal tumors (GIST): analyses from INVICTUS. J Clin Oncol.

[44] Blay JY, Le Cesne A, Ray-Coquard I (2007). Prospective multicentric randomized phase III study of imatinib in patients with advanced gastrointestinal stromal tumors comparing interruption versus continuation of treatment beyond 1 year: the French Sarcoma Group. J Clin Oncol.

[45] Le Cesne A, Ray-Coquard I, Bui BN (2010). Discontinuation of imatinib in patients with advanced gastrointestinal stromal tumours after 3 years of treatment: an open-label multicentre randomised phase 3 trial. Lancet Oncol.

[46] Eichler M, Pink D, Menge F (2021). Quality of life of GIST patients with and without current tyrosine kinase inhibitor treatment: cross-sectional results of a German multicentre observational study (PROSa). Eur J Cancer Care (Engl).

