# Supplementary material for: The GIST of it all: management of gastrointestinal stromal tumors (GIST) from the first steps to tailored therapy. A bibliometric analysis
Source: Langenbecks Arch Surg. 2024 Mar 14;409(1):95. doi: 10.1007/s00423-024-03271-6 (PMC10937785; doi:10.1007/s00423-024-03271-6)

Supplementary Figure 1 Musa *et al.* 2023

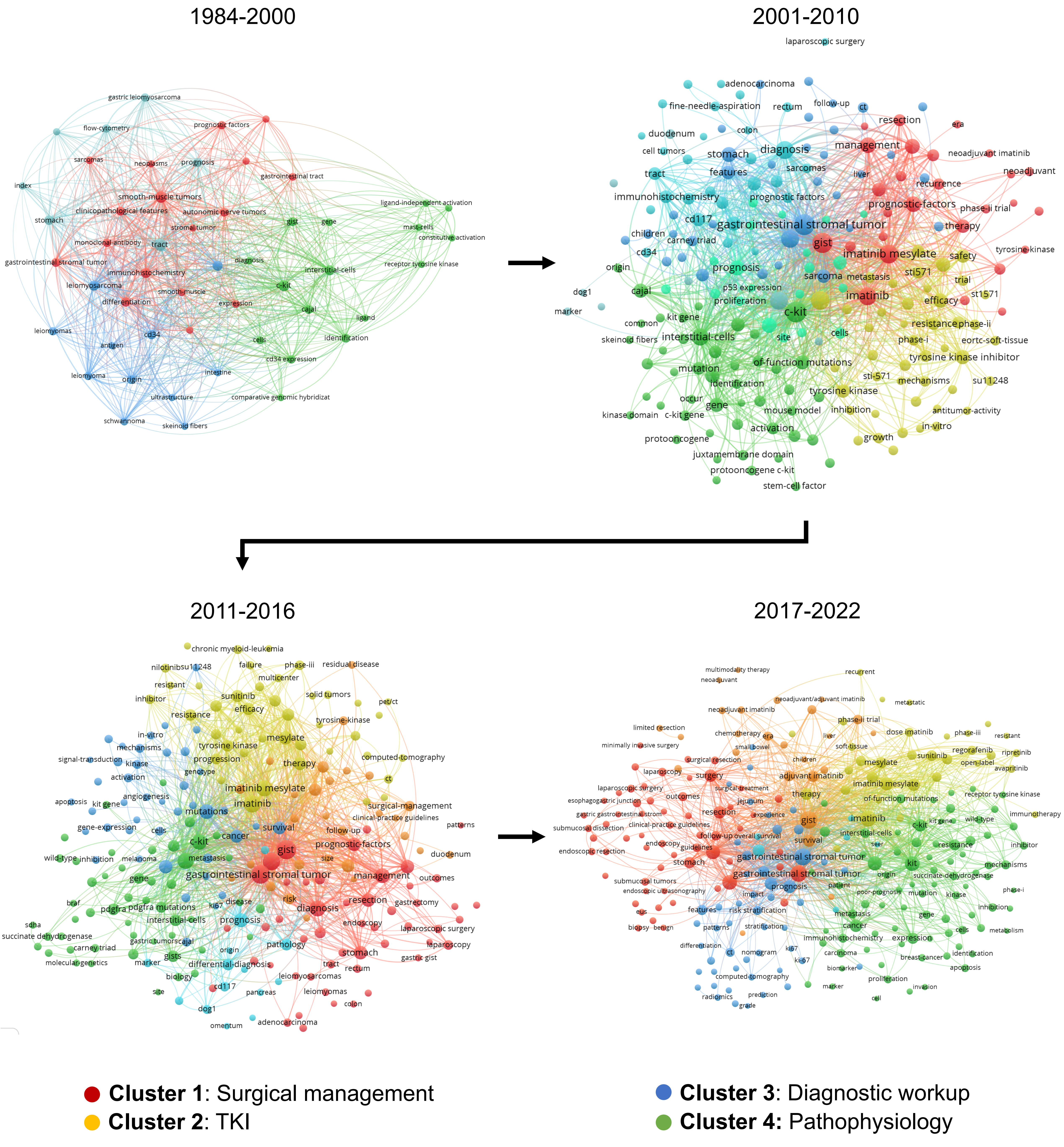

Supplement: Supplementary file 1 — Supplementary file1 (PDF 7050 KB) [file 423_2024_3271_MOESM1_ESM.pdf]
